# 309. TB Quantiferon Testing Predicts Mortality in Patients with COVID-19

**DOI:** 10.1093/ofid/ofab466.511

**Published:** 2021-12-04

**Authors:** Mirza Z Baig, Siyun Liao, Margaret Powers-Fletcher, Moises A Huaman, Senu Apewokin

**Affiliations:** 1 University of Cincinnati, Mason, Ohio; 2 UC Health-University of Cincinnati Medical Center, Cincinnati, OH

## Abstract

**Background:**

Finding reliable clinical predictors for severity of COVID-19 has been challenging. Interferon gamma (IFNG) plays an important role in viral replication. QuantiFERON-TB (QFT) test relies on IFNG release in response to antigens. A positive or negative test signifies adequate IFNG response, whereas an indeterminate result is obtained when such a response is lacking. In this study, we have attempted to see if an indeterminate QFT result can provide prognostic information on patients with COVID–19.

Survival Probability in patients with Covid - 19 and an indeterminate TB Quantiferon test result

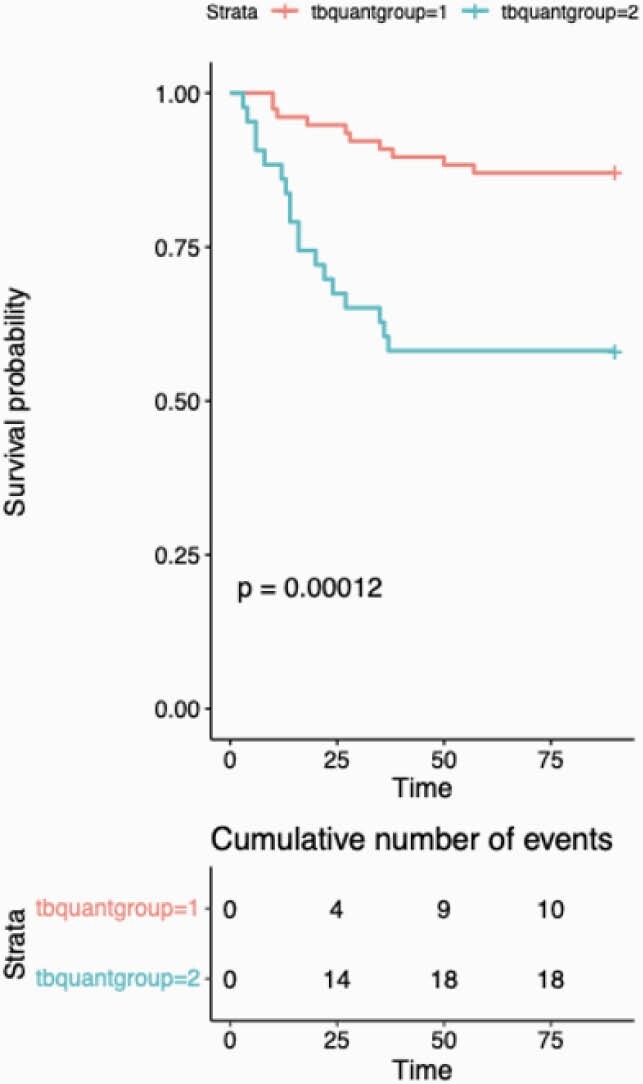

**Methods:**

This is a retrospective study of patients who were admitted at our institute with COVID–19 and had a QFT done within one month of the positive SARS-CoV-2 nucleic acid amplification test result. Patient charts were analyzed for clinical course and outcomes, including in-hospital mortality (primary outcome), 90-day mortality, respiratory failure, requirement for intubation and other complications that would portend a more severe disease course.

**Results:**

A total of 120 patient charts were analyzed, out of which 43 (35.8%) had an indeterminate QFT. All the indeterminate results were due to an inadequate mitogen response. The indeterminate QFT group had a 41.86% (18/43) in-hospital mortality vs. 9.09% (7/77) in the negative or positive QFT group (p-value of < 0.001). The 90-day mortality was similar between the two groups. Patients with indeterminate QFT also had a higher incidence of respiratory failure (97.7% vs. 75.3%; p-value = 0.020), requirement for mechanical ventilation (55.8% vs. 23.4%; p-value < 0.001), requirement of ECMO (25.58% vs. 0%; p-vale < 0.001), requirement of pressor (48.83% vs. 14.28%; p-value < 0.001) and requirement for renal replacement therapy (32.5% vs. 1.3%; p-value < 0.001), when compared to patients with a negative or positive QFT. Patients in indeterminate group had a higher hospital length of stay than the other group (p-value = 0.035).

**Conclusion:**

Our study indicates that patients with COVID-19 who fail to mount an adequate IFNG mitogen response in QFT assay have worse clinical outcomes and a more complicated and protracted clinical course. Evaluating cell-mediated immune responses through commercially available IFNG release assays may yield a promising strategy to predict COVID-19 clinical outcomes.

**Disclosures:**

**All Authors**: No reported disclosures

